# Screening and bioinformatical analysis of differentially expressed genes in nasopharyngeal carcinoma

**DOI:** 10.7150/jca.48979

**Published:** 2021-01-30

**Authors:** Weiqian Guo, Xiaomin Zheng, Lei Hua, Xianbin Zheng, Yangyang Zhang, Bin Sun, Zhenchao Tao, Jin Gao

**Affiliations:** 1 Department of Radiation Oncology, Anhui Provincial Hospital Affiliated to Anhui Medical University, Hefei 230001, China.; 2 Xinxiang Medical University, Xinxiang 453000, China.; 3 Department of Radiation Oncology, the First Affiliated Hospital of USTC, Division of Life Sciences and Medicine, University of Science and Technology of China, Hefei 230031, China.

**Keywords:** nasopharyngeal carcinoma, core genes, bioinformatical analysis, prognosis, microarray

## Abstract

**Objective:** To identify differentially expressed genes via bioinformatical analysis for nasopharyngeal carcinoma (NPC) and explore potential biomarkers for NPC.

**Methods:** We downloaded the NPC gene expression datasets (GSE40290, GSE53819) and obtained differentially expressed genes (DEGs) via GEO2R. Functional analysis of DEGs was performed by Gene Ontology (GO) and Kyoto Encyclopedia of Genes and Genomes (KEGG) analysis. In order to explore the interaction of DEGs and screen the core genes, we established protein-protein interaction (PPI) network. Then the expression level, prognostic and diagnostic analysis of the core genes in NPC were performed to reveal their potential effects on NPC. Furthermore, we obtained the transcription factors (TF) and microRNAs of core genes to construct the coregulatory network.

**Results:** We obtained 124 up-regulated genes and 190 down-regulated genes in total. These genes were found to be related to signal transduction, extracellular matrix organization and cell adhesion based on GO analysis. KEGG analysis revealed that the NF-kappa B (NF-κB) signaling pathway, pathways in cancer were mainly enriched signaling pathways. 25 core genes were obtained by constructing PPI network. Then the high expression of 10 core genes in NPC were verified via GEPIA, Oncomine databases and laboratory experiments. The TF-microRNA coregulatory network of the 10 core genes was built. Survival and diagnostic analysis indicated that SPP1 had negative influence on the prognosis of NPC patients based on two datasets and nine up-regulated core genes (FN1, MMP1, MMP3, PLAU, PLAUR, SERPINE1, SPP1, COL8A1, COL10A1) might be diagnostic markers for NPC.

**Conclusions:** Core genes of NPC were screened out by bioinformatical analysis in the present study and these genes may serve as prognostic and diagnostic biomarkers for NPC.

## Introduction

Nasopharyngeal carcinoma (NPC) is a malignancy in head and neck arising from the nasopharyngeal epithelium 1. NPC has a unique geographical distribution, which the incidence of NPC in Southeast Asia is higher than that in western countries 2. 86000 new cases of NPC were reported globally in 2012 and there were approximately 50000 patients died 3. Multiple factors participate in the pathogenesis of NPC, including the genetic factors, dietary factors, Epstein-Barr virus 4, 5. NPC is sensitive to radiotherapy and because of its deep anatomical position, which limits the operation; chemoradiotherapy has become the main treatment of NPC. After treatment, the 5-year survival rate of patients with early NPC can be more than 90% 6, but local recurrence and metastasis greatly affect the prognosis of patients 7. Thus, there is an urgent need to explore pathogenesis of NPC and find the early diagnostic markers for NPC.

With the development of molecular biotechnology, bioinformatical analysis plays an important role in screening tumor candidate biomarkers, and it provides new clues for the study of tumor pathogenesis including NPC 8. For example, Zhu et al. confirmed that the curcumin could improve radiosensitization of NPC cell lines by constructing a competing endogenous RNA network based on bioinformatical analysis 9. Zhang et al. screened several serum microRNAs as potential prognostic markers of NPC patients with concurrent chemoradiotherapy by microRNA chip and bioinformatical analysis 10. Although many studies have been carried out for NPC, there is still a lack of biomarkers for prognosis and early diagnosis of NPC and further studies to explore therapeutic targets for NPC are still needed.

In the current study, we obtained GSE40290 and GSE53819 from the Gene Expression Omnibus (GEO) database. Differentially expressed genes (DEGs) were screened out by GEO2R online tool. The Gene Ontology (GO) terms and Kyoto Encyclopedia of Genes and Genomes (KEGG) pathway analysis were carried out for better understanding of DEGs. Subsequently, core genes were obtained by constructing protein-protein interaction (PPI) network. The expression of core genes in NPC were validated by Gene Expression Profiling Interactive Analysis (GEPIA), Oncomine databases and laboratory experiments. Prognostic analysis revealed that SPP1 had negative influence on the overall survival and progression-free survival (PFS) of NPC patients and diagnostic analysis indicated that nine core genes might be diagnostic markers for NPC. In conclusion, we identified potential biomarkers of NPC based on bioinformatical analysis, which might be targets of treatment for NPC patients.

## Materials and Methods

### Information of datasets

We searched gene expression datasets of NPC from GEO database (http://www.ncbi.nlm.nih.gov/ geo/). The inclusion criteria were as follows: (1) the datasets of mRNA expression profile, (2) the datasets of NPC tissues and normal nasopharyngeal tissues, (3) the datasets contained more than five pairs of NPC tissues and normal tissues, (4) all NPC tissues and normal tissues were confirmed by histopathology. The datasets that did not meet these criteria were excluded. Then, two datasets (GSE40290 and GSE53819) were obtained. The GSE40290 dataset contained 25 NPC tissues and 8 normal nasopharyngeal tissues, which was detected by GPL8380 platform (Capitalbio 22K Human oligo array version 1.0). The GSE53819 dataset contained 18 NPC tissues and 18 normal nasopharyngeal tissues, which was detected by GPL6480 platform (Agilent-014850 Whole Human Genome Microarray 4x44K G4112F). Then, GSE102349 with 113 NPC patients and GSE12452 with 31 NPC patients were also downloaded for survival and diagnostic analysis respectively, because of their completely clinical information.

### Acquisition of differentially expressed genes (DEGs)

GEO2R was applied to identify the DEGs of GSE40290 and GSE53819 according to the criteria of adjusted* P* value < 0.05 and | logFC | > 1. The genes with logFC > 1 were considered as up-regulated genes and the genes with logFC < -1 were considered as down-regulated genes. Then the heat map of top 50 DEGs and the volcano map of all genes were drawn by pheatmap and ggplot2 package of R 3.6.1 software. Venn software was used to obtain the overlap of the two datasets.

### Functional annotation of DEGs

GO analysis interpreted genes from three aspects: biological process (BP), cellular component (CC) and molecular function (MF). Signaling pathways, which the DEGs might involve, were analyzed via the KEGG pathway analysis. GO terms and KEGG pathway analysis of DEGs were carried out through the Database for Annotation, Visualization and Integrated Discovery (DAVID, https://david.ncifcrf.gov/) and the cut-off criterion of statistical significance was *P <* 0.05. Then R software was used to show the results.

### Screening of core genes by constructing PPI network

In order to explore the interaction of DEGs, we constructed PPI network by the Search Tool for the Retrieval of Interacting Genes (STRING, http://www.string‑db.org/) and Cytoscape 3.6.1 was used to show the network. The MCODE app of Cytoscape was applied to obtain the core genes and the parameters of MCODE were set to node score cutoff = 0.2, degree cutoff = 2, max. depth = 100 and k-core = 2. The genes with the highest MCODE-score were regarded as core genes. Then the biological process that core genes might involve was predicted and visualized by the BinGO app in Cytoscape software (*P* < 0.01).

### Validation of expression and clinical analysis of core genes

The GEPIA (http://gepia.cancer-pku.cn/) and the Oncomine databases (https://www.oncomine.org) were applied to validate the expression level of core genes between NPC tissues and normal nasopharyngeal tissues. The effect of core genes on survival time of NPC patients was showed by GEPIA database. Then GSE102349 dataset was used to evaluate the effect of core genes on PFS of NPC patients via R software and GSE12452 dataset was used to evaluate the diagnostic value of these core genes for NPC with receiver operating characteristic (ROC) curve via GraphPad Prism software. cBioPortal database (https://www.cbioportal.org/) was applied to perform analysis of genetic alterations related to the core genes based on TCGA. The cut-off criterion of statistical significance was *P <* 0.05.

### TF-microRNA coregulatory interactions

The NetworkAnalyst (https://www.networkanalyst.ca/NetworkAnalyst/home.xhtml) is an online analytic platform. The transcription factors (TF) and microRNAs of core genes were obtained via the NetworkAnalyst online tool based on RegNetwork repository, which collected the literature curated regulatory interaction information. The coregulatory network was constructed by Cytoscape software. Then, the expression of key elements of the network in NPC cells were evaluated by qRT-PCR.

### Cell lines

Human NPC cell lines (CNE1, CNE2, 6-10B, 5-8F) and normal nasopharyngeal cell (NP69) were from Sun Yat-sen University (Guangzhou, China). They were cultured in RPMI-1640 (Biological Industries) with 10% fetal bovine serum (Biological Industries) and stored at 37℃ with 5% CO_2_.

### RNA extraction and quantitative real-time PCR (qRT-PCR)

Total RNAs of cells were extracted by Trizol reagent (Invitrogen) and converted into complementary DNA (cDNA) using the PrimeScript^TM^RT Master Mix (Takara, China) according to the manufacture's protocol. Then, TB Green^®^ Premix Ex Taq^TM^ II (Takara, China) was used to perform qRT-PCR. GAPDH and U6 was used as an internal control to normalize results and the relative expression of core genes were evaluated by the 2^-ΔΔCt^ method. All experiments were repeated in three times. The primers were synthesized by Sango Biotech (Shanghai, China) and the sequences of all primers were presented in Table 1.

### Western blot analysis

Three core genes (COL8A1, COL10A1, COL17A1), that have not been reported in NPC, were selected for western blot analysis in cell lines. Cells were collected at 90% confluence and total proteins of cells were extracted with cell lysis buffer and heated at 95 °C for 10 minutes. Then the proteins were electrophoresed and transferred to polyvinyl fluoride (PVDF) membranes (Immobilon®-P). After sealing with 5% defatted milk for 2h at room temperature (RT), the membranes were incubated with the first antibody overnight at 4℃. Then the membranes were incubated with the second antibody for 2h at RT after washing with PBST. The target bands of the membrane were visualized and photographed by smearing the chemiluminescence reagent. Then, Image J software was used to quantify the relative intensity level of the target bands. The anti-COL8A1 (ab58776), anti-COL10A1 (ab58632) and anti-COL17A1 (ab184996) were purchased from Abcam. The anti-GAPDH (10494-1-AP), HRP goat anti-rabbit IgG antibody (SA00001-2) were provided by San Ying Biotechnology, China.

### Statistical analysis

GraphPad Prism 8 and Excel were used for statistical analysis. The results were described as mean ± SD. Two-tailed Student's *t*-test was used to calculate statistical significance. The cut-off criterion of statistical significance was *P <* 0.05.

## Results

### Screening of DEGs in NPC

**Figure 1:**
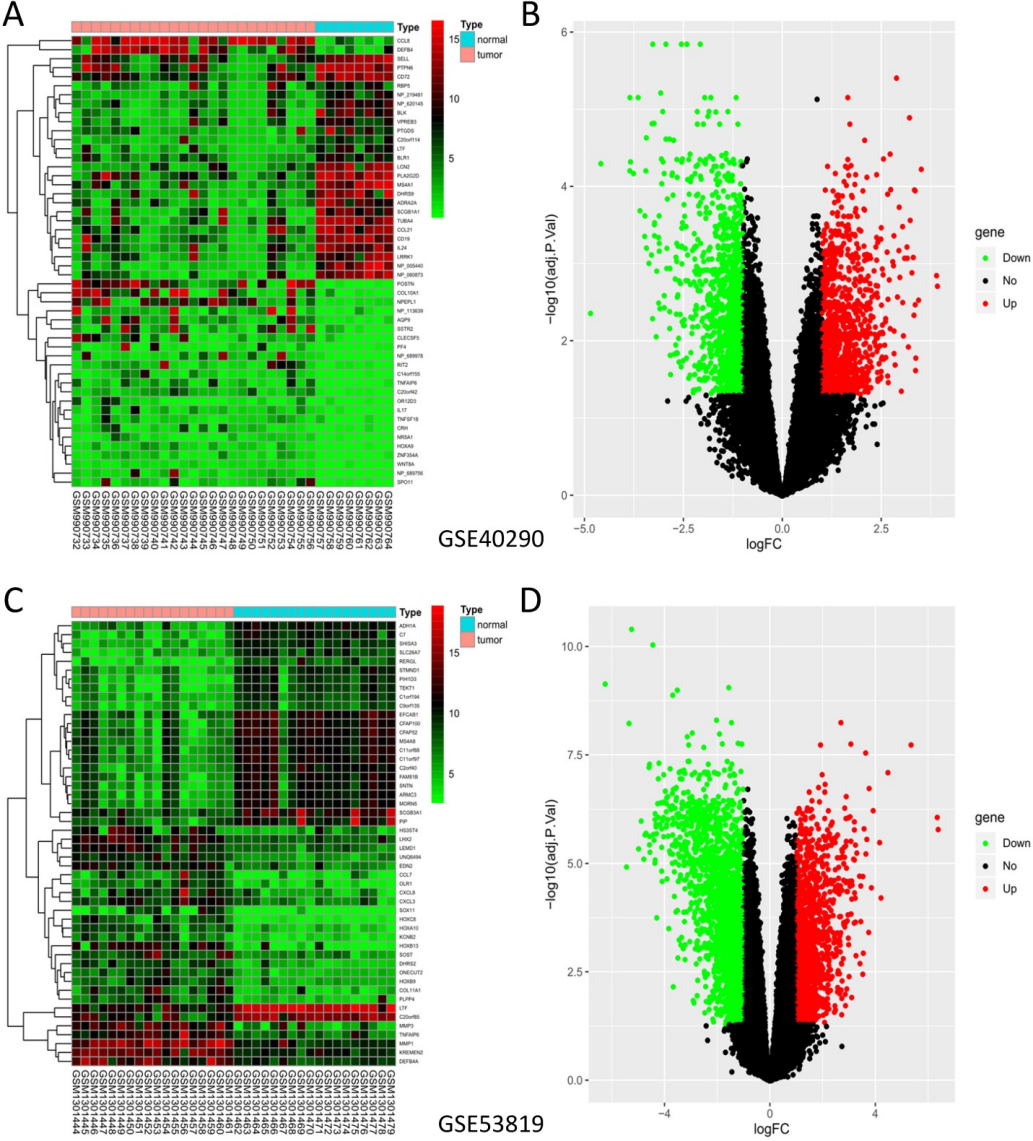
DEGs of GSE40290 and GSE53819. Heatmap of the top 50 DEGs: **(A)** GSE40290; **(C)** GSE53819; Volcano map: **(B)** GSE40290; **(D)** GSE53819. Red: up-regulated DEGs, green: down-regulated DEGs, black: no difference.

**Figure 2:**
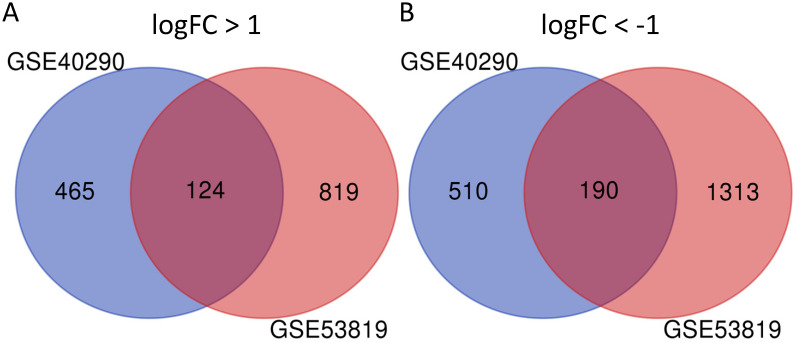
314 commonly DEGs of GSE40290 and GSE53819. **(A)** 124 up-regulated DEGs; **(B)** 190 down-regulated DEGs.

GSE40290 and GSE53819 contained 43 NPC tissues and 26 normal nasopharyngeal tissues totally. On the basis of selection criteria including adjusted* P* value < 0.05, | logFC | > 1 via GEO2R tool, 1289 DEGs were identified in GSE40290 totally, including 589 up-regulated genes and 700 down-regulated genes (Fig. 1B). Then 2446 DEGs were identified in GSE53819 totally, including 943 up-regulated genes and 1503 down-regulated genes (Fig. 1D). The heat maps showed the top 50 DEGs of GSE40290 and GSE53819 (Fig. 1A, C). Then, the overlap of GSE40290 and GSE53819 was obtained by Venn software, which contained 314 commonly DEGs including 124 up-regulated genes and 190 down-regulated genes (Fig. 2A, B).

### Functional analysis of DEGs

**Figure 3:**
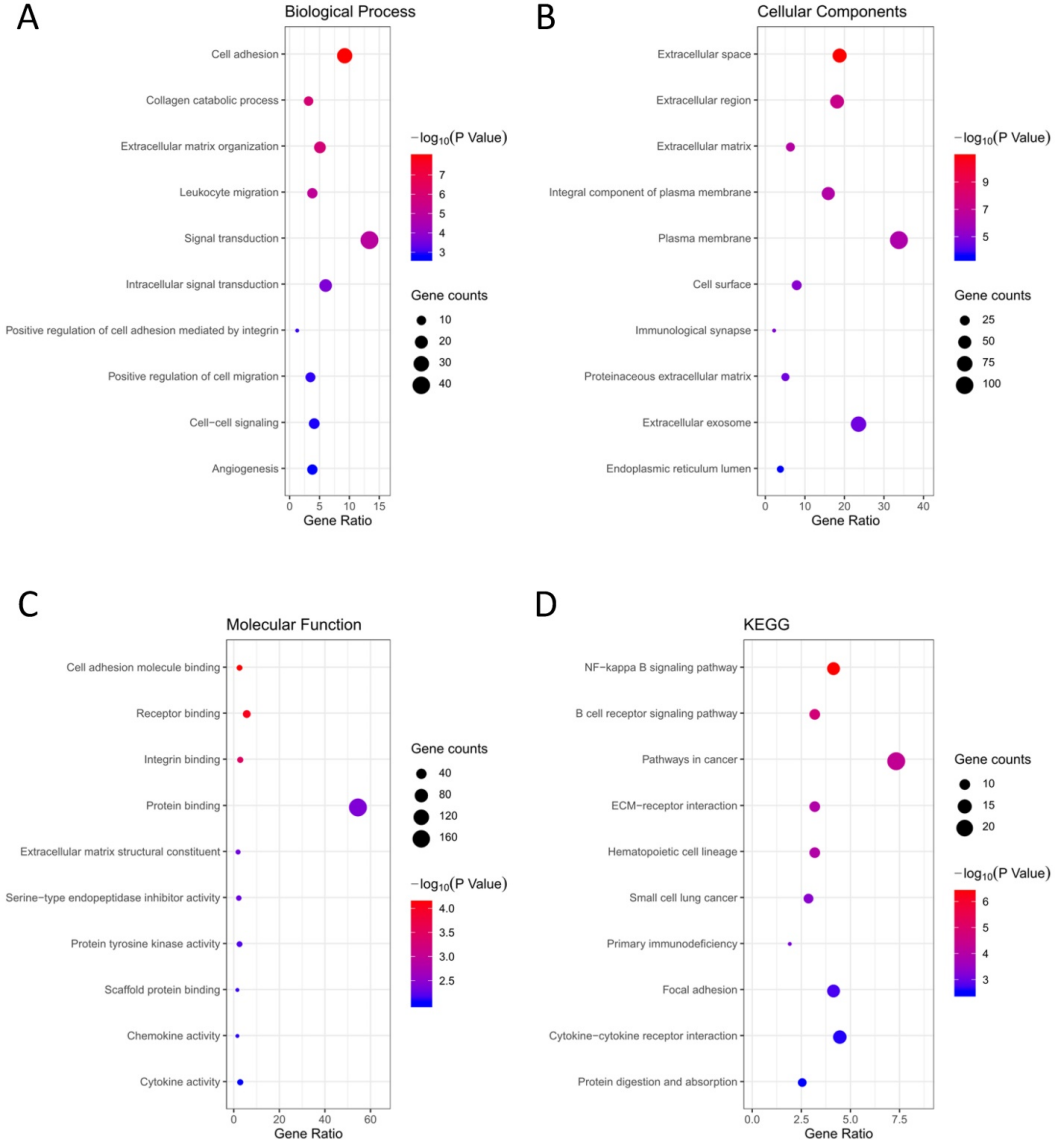
Functional analysis of the 314 DEGs. **(A)** BP; **(B)** CC; **(C)** MF; **(D)** KEGG pathway analysis.

GO terms and KEGG pathway analysis of the 314 DEGs were carried out via DAVID database. BP, CC and MF are parts of GO annotation. For BP, the DEGs were mainly related to signal transduction, extracellular matrix organization, cell adhesion, collagen catabolic process and leukocyte migration, et al. (Fig. 3A, Table 2). For CC, the DEGs were mainly related to extracellular region, plasma membrane, extracellular space, extracellular matrix and integral component of plasma membrane, et al. (Fig. 3B, Table 2). For MF, the DEGs were mainly related to protein binding, cell adhesion molecule binding, receptor binding, integrin binding and extracellular matrix structural constituent, et al. (Fig. 3C, Table 2). KEGG analysis revealed that the NF-kappa B (NF-κB) signaling pathway, pathways in cancer, B cell receptor signaling pathway, ECM-receptor interaction and hematopoietic cell lineage were mainly enriched signaling pathways (Fig. 3D, Table 2).

### Screening of core genes based on PPI network

**Figure 4:**
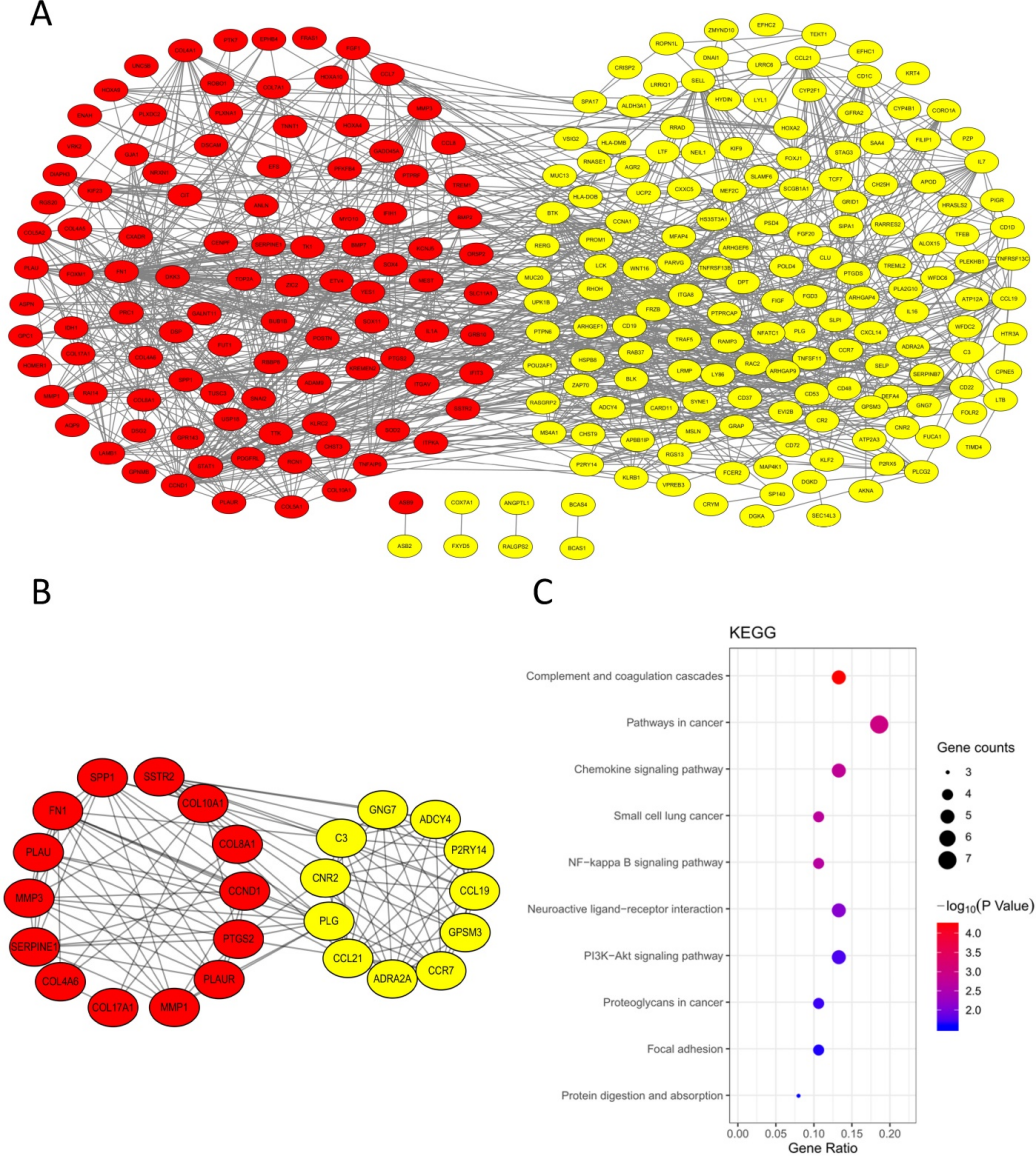
PPI network. **(A)** PPI network of the 314 DEGs; **(B)** Module analysis of the network. Red: up-regulated genes, yellow: down-regulated genes; **(C)** KEGG analysis of the 25 core genes in the module.

In order to explore the interaction of 314 DEGs, we established PPI network. As shown, the network contained 313 nodes and 1026 edges (Fig. 4A). Subsequently, the module with the highest MCODE-score (9.583) was screened out by MCODE app and the module contained 25 core genes and 115 edges (Fig. 4B). The KEGG pathway of 25 core genes was analyzed by DAVID and they were mainly enriched in pathways in cancer, small cell lung cancer, complement and coagulation cascades, chemokine signaling pathway and NF-κB signaling pathway, et al. (Fig. 4C, Table 3).

### The expression of core genes

**Figure 5:**
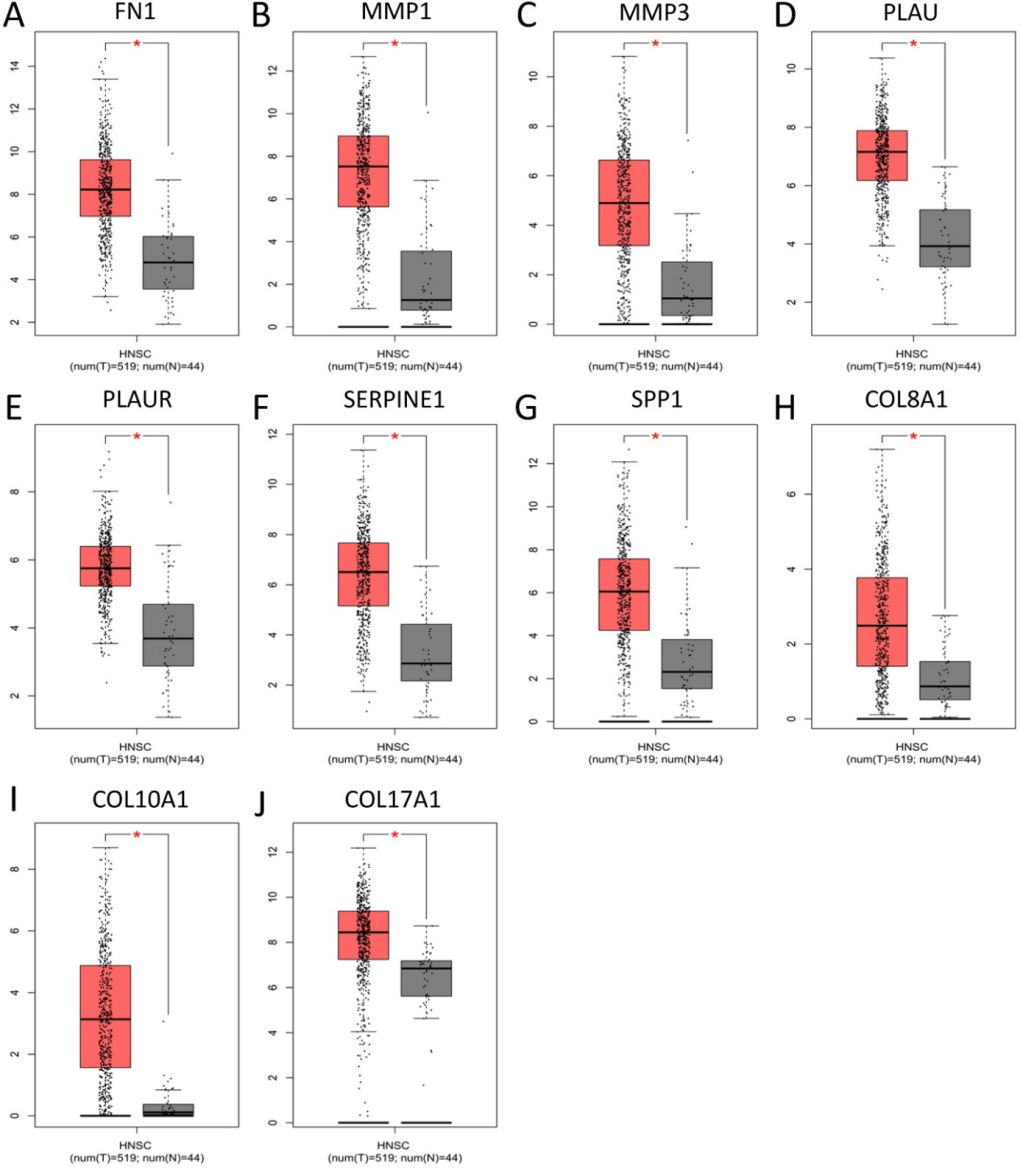
Significantly expressed 10 core genes in NPC tissues compared to normal tissues in GEPIA: **(A)** FN1; **(B)** MMP1;** (C)** MMP3; **(D)** PLAU; **(E)** PLAUR; **(F)** SERPINE1; **(G)** SPP1; **(H)** COL8A1; **(I)** COL10A1; **(J)** COL17A1. Red: tumor tissues, grey: normal tissues. *: *P* < 0.05.

**Figure 6:**
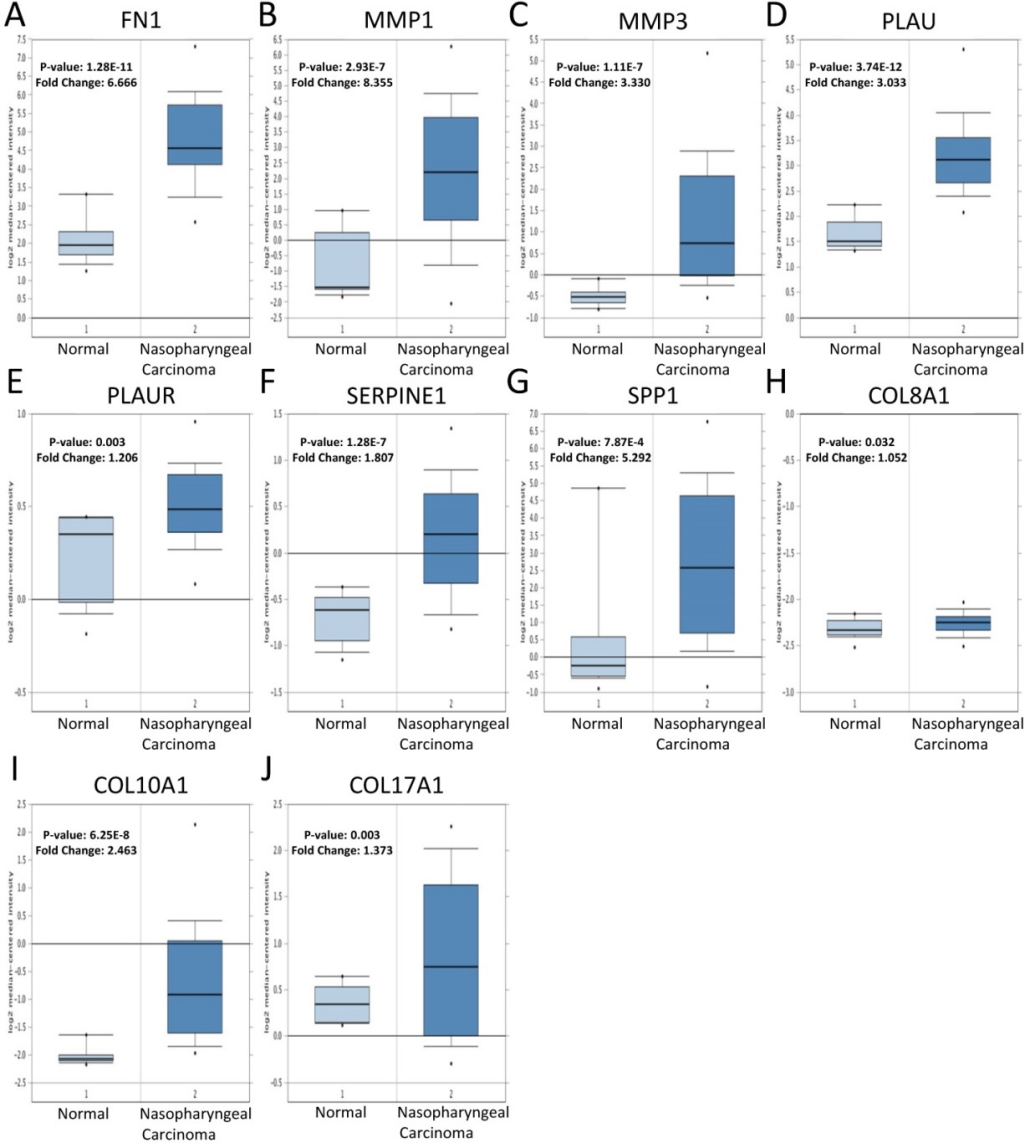
Verification of the expression of the 10 core genes in NPC tissues compared to normal tissues in Oncomine: **(A)** FN1;** (B)** MMP1; **(C)** MMP3; **(D)** PLAU; **(E)** PLAUR; **(F)** SERPINE1; **(G)** SPP1; **(H)** COL8A1; **(I)** COL10A1; **(J)** COL17A1. The cut-off criterion of statistical significance was *P* < 0.05.

To validate the expression of 25 core genes in NPC tissues, we uploaded the 25 core genes to GEPIA database and there were 10 core genes were revealed to be significantly over-expressed in NPC tissues contrasted to normal nasopharyngeal tissues, including FN1, MMP1, MMP3, PLAU, PLAUR, SERPINE1, SPP1, COL8A1, COL10A1 and COL17A1, which were consistent with GSE40290 and GSE53819 (*P* < 0.05, Fig. 5). The results were also validated by Oncomine database (FN1: fold change = 6.666, *P* = 1.28E-11; MMP1: fold change = 8.355, *P* = 2.93E-7; MMP3: fold change = 3.330, *P* = 1.11E-7; PLAU: fold change = 3.033, *P* = 3.74E-12; PLAUR: fold change = 1.206, *P* = 0.003; SERPINE1: fold change = 1.807, *P* = 1.28E-7; SPP1: fold change = 5.292, *P* = 7.87E-4; COL8A1: fold change = 1.052, *P* = 0.032; COL10A1: fold change = 2.463, *P* = 6.25E-8; COL17A1: fold change = 1.373, *P* = 0.003) (Fig. 6).

### Experimental validation

**Figure 7:**
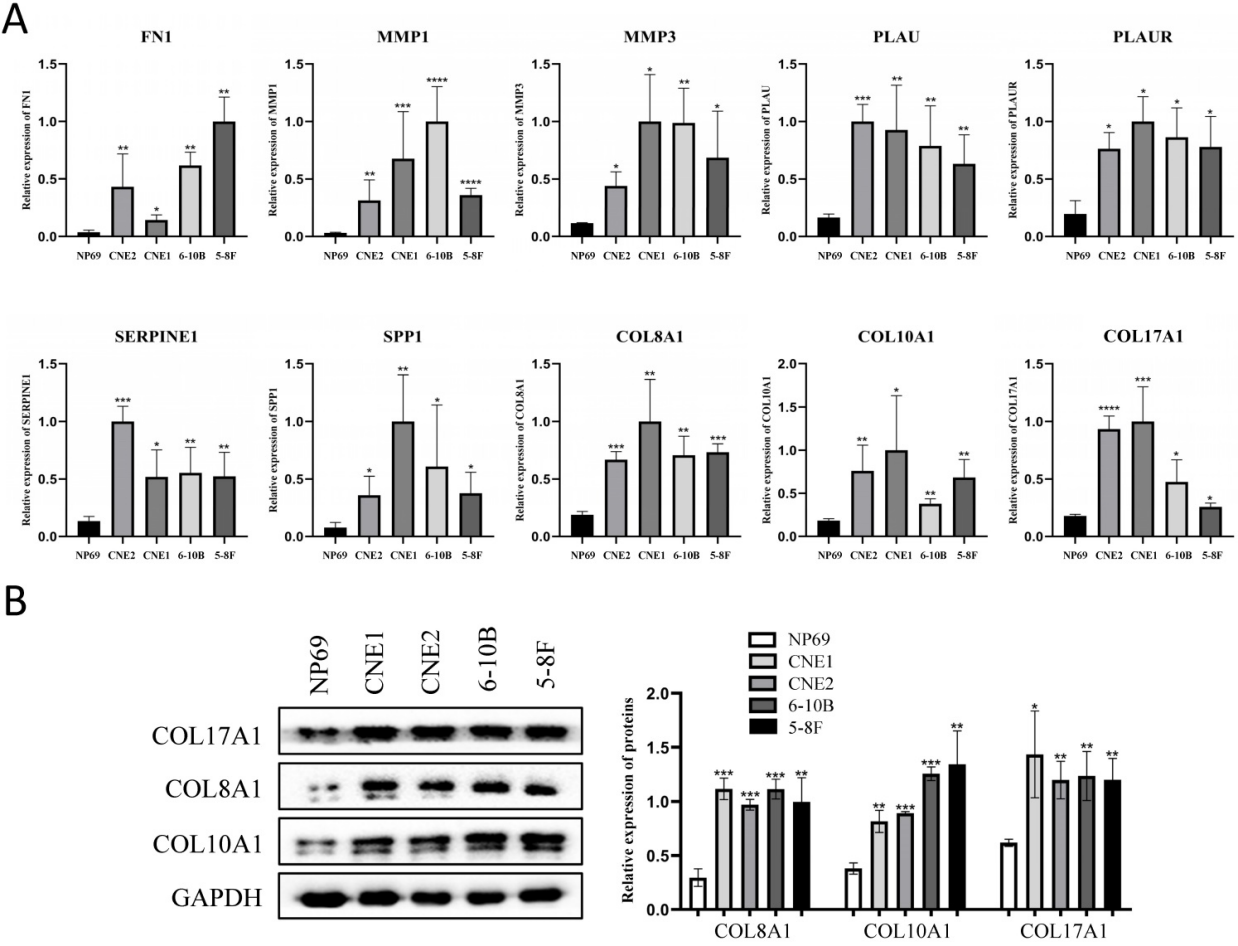
The expression of the 10 core genes in NPC cells (CNE1, CNE2, 6-10B, 5-8F) and normal nasopharyngeal cell (NP69). **(A)** qRT-PCR; **(B)** Western blot analysis. *: *P* < 0.05, **: *P* < 0.01, ***: *P* < 0.001, ****: *P* < 0.0001.

The expression of the 10 core genes in NPC cells (CNE1, CNE2, 6-10B, 5-8F) and normal nasopharyngeal cell (NP69) were evaluated by qRT-PCR and we found that they were significantly up-regulated in NPC cells compared with normal nasopharyngeal cell (*P* < 0.05, Fig. 7A).

Furthermore, the roles of COL8A1, COL10A1 and COL17A1 in NPC have not been reported, while other seven core genes in NPC have been studied already. Therefore, they were selected for western blot analysis in cell lines and they were also validated to be up-regulated in NPC cells at protein level (*P* < 0.05, Fig. 7B).

### Genetic information of core genes

**Figure 8:**
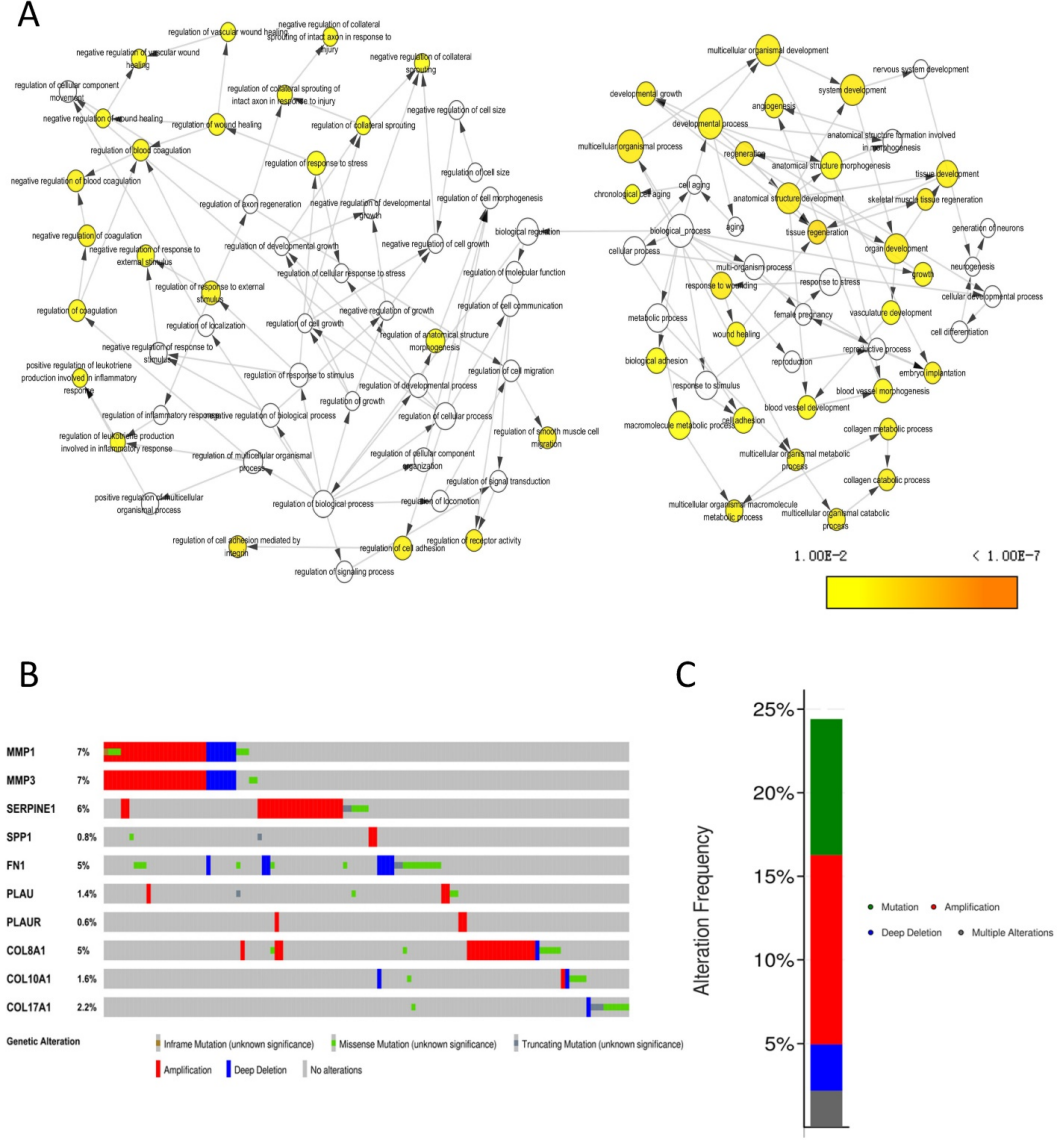
Genetic information of the 10 core genes. **(A)** Biological process (*P* < 0.01); **(B)** Genetic alterations in cBioPortal database; **(C)** Alteration frequency.

The biological process that the 10 core genes might involve was analyzed by the BinGO app of Cytoscape software and they were enriched in multicellular organismal development, angiogenesis and regulation of wound healing, et al. (Fig. 8A). By analyzing genetic alterations of the 10 core genes in NPC via cBioPortal database, we found that MMP1 (7%), MMP3 (7%) and SERPINE1 (6%) had the highest frequency of alterations in the 10 core genes (Fig. 8B) and the alterations of the 10 core genes occurred in 123 (24%) of 504 patients (Fig. 8C).

### The construction of TF-microRNA coregulatory network

**Figure 9:**
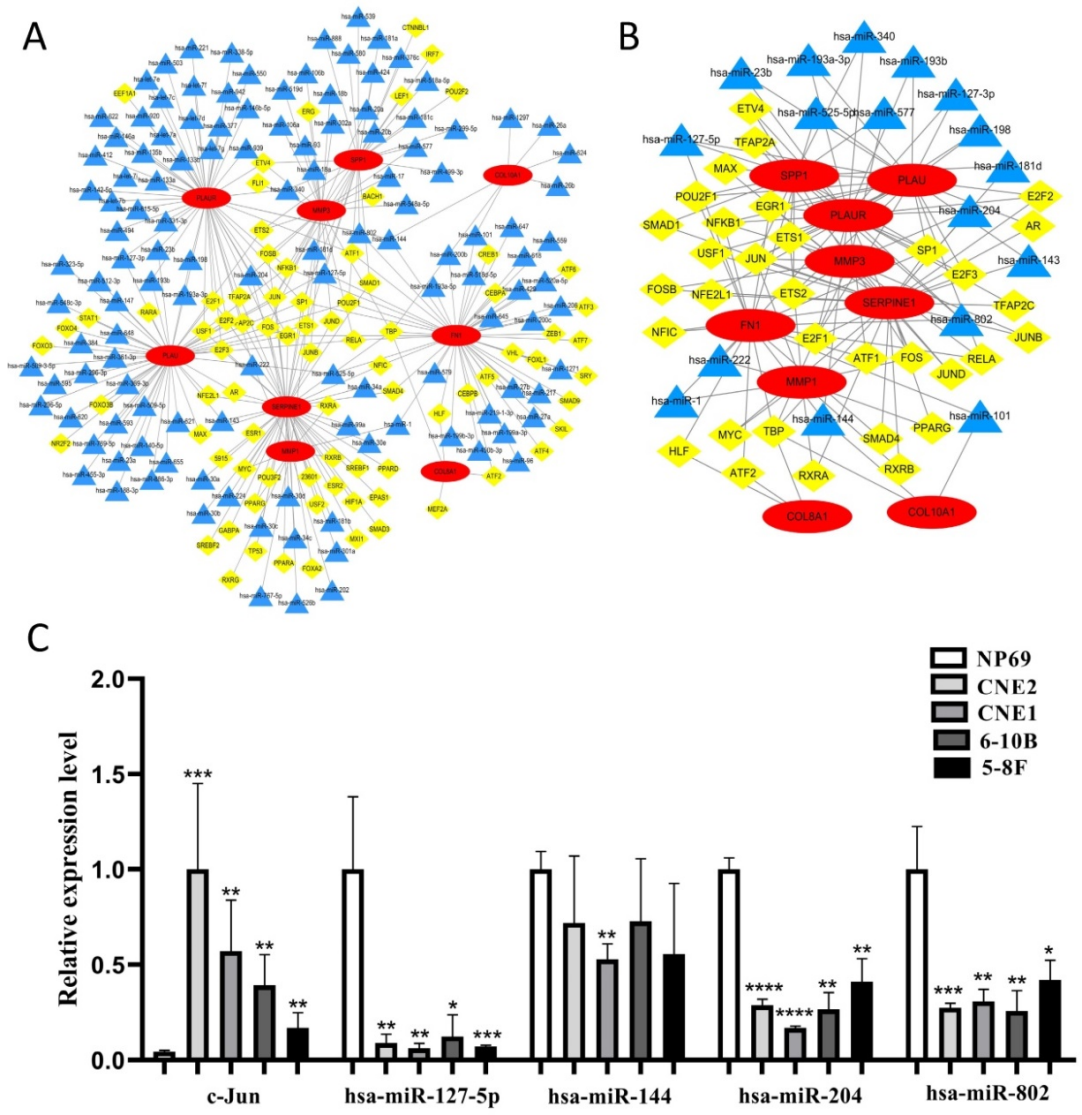
The construction of TF-microRNA coregulatory network. **(A)** Integrative regulatory network of TF-mRNA-microRNA; **(B)** Network after removing the points with only one degree of correlation; **(C)** The expression of key elements in TF-microRNA coregulatory network in NPC cells. Red: core genes, yellow: TFs, blue: microRNAs, *: *P* < 0.05, **: *P* < 0.01, ***: *P* < 0.001, ****: *P* < 0.0001.

We constructed the TF-microRNA coregulatory network of 10 core genes by NetworkAnalyst database and Cytoscape software to further investigate the potential function of the 10 core genes. In the network, nine core genes could be identified (except for COL17A1) and there were 80 TFs, 127 microRNAs totally (Fig. 9A). After removing the points with only one degree of correlation, 33 TFs and 17 microRNAs were identified (Fig. 9B). We found that Jun could regulate six core genes. Simultaneously, four microRNAs (hsa-miR-127-5p, hsa-miR-144, hsa-miR-204, hsa-miR-802) could regulate three core genes. Therefore, Jun and the four microRNAs were selected as key elements in the network and their expression in NPC cells were evaluated by qRT-PCR (Fig. 9C). We found that Jun was significantly up-regulated in NPC cells, and three microRNAs (hsa-miR-127-5p, hsa-miR-204, hsa-miR-802) were significantly down-regulated in NPC cells (*P* < 0.05). These results indicated that the key elements might be involved in the upstream mechanisms that regulate core genes.

### Clinical analysis of core genes

**Figure 10:**
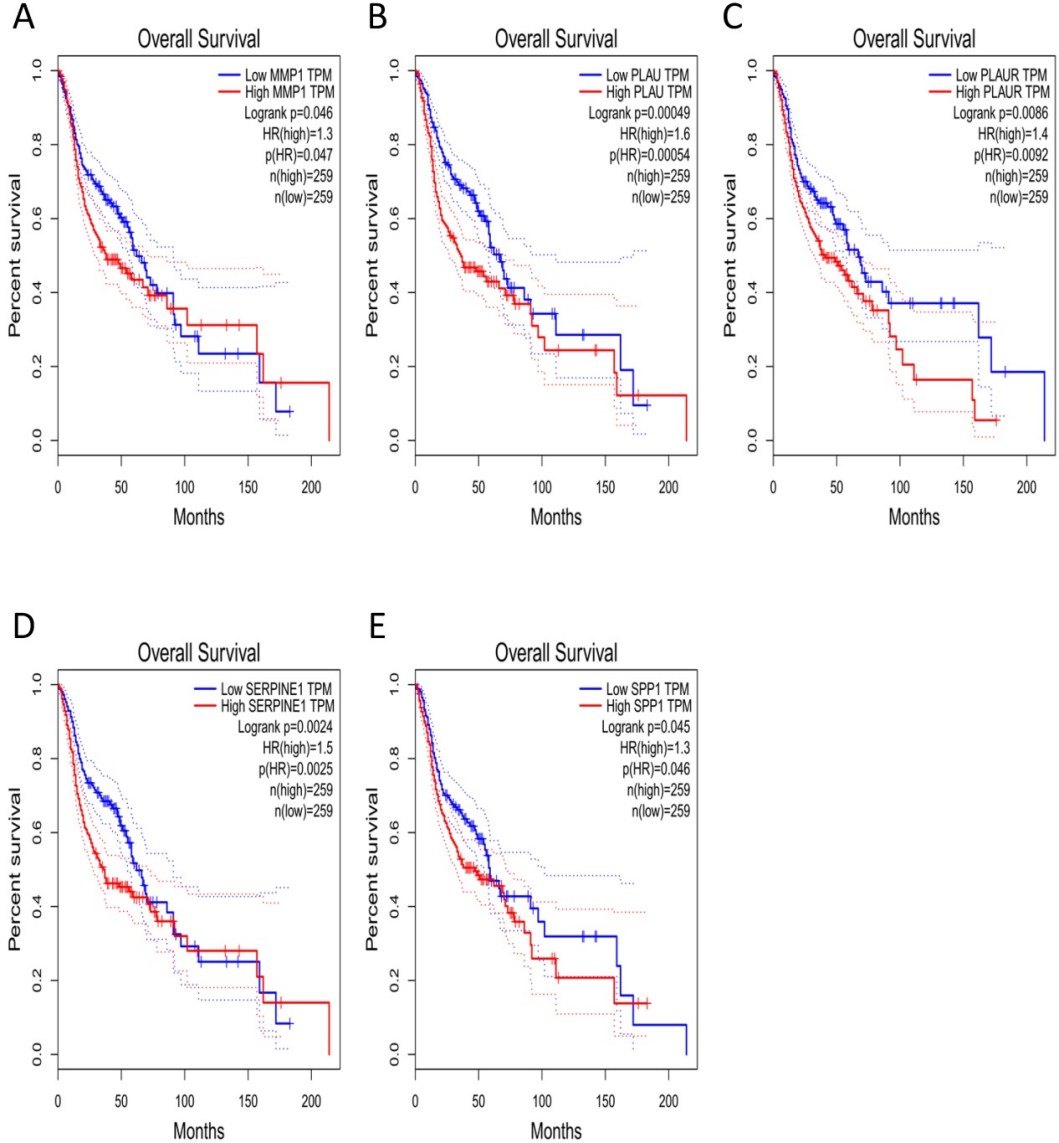
The prognostic analysis of core genes in NPC based on GEPIA: **(A)** MMP1; **(B)** PLAU; **(C)** PLAUR; **(D)** SERPINE1;** (E)** SPP1. The cut-off criterion of statistical significance was *P* < 0.05.

**Figure 11:**
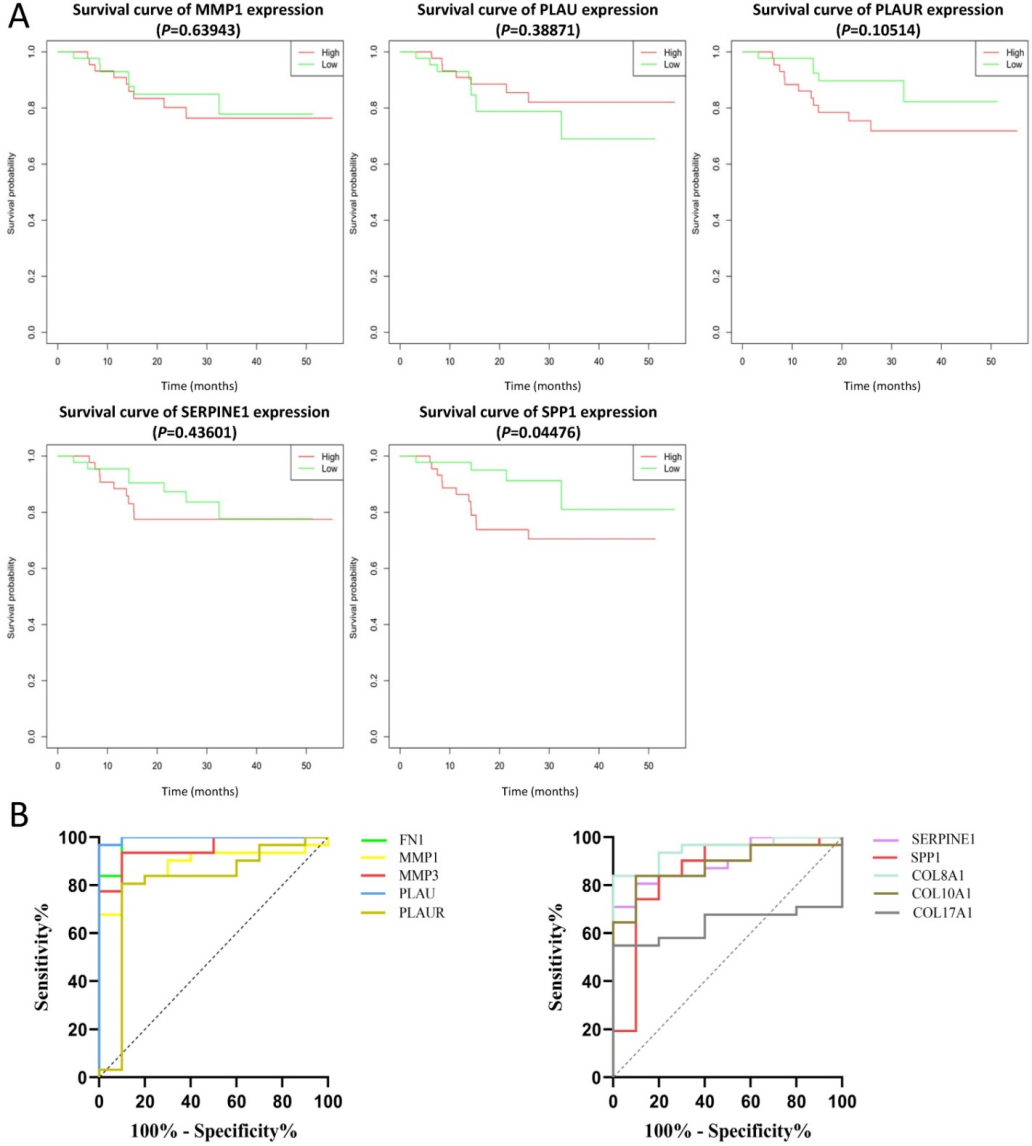
The prognostic analysis **(A)** and diagnostic analysis **(B)** of core genes in NPC based on GSE102349 and GSE12452 datasets, respectively. The cut-off criterion of statistical significance was *P* < 0.05.

The effect of the 10 core genes on prognosis of NPC patients was analyzed by GEPIA database. Then we found that 5 of the 10 core genes had influence on the survival of NPC patients, including MMP1 (HR = 1.3, *P* = 0.04700), PLAU (HR = 1.6, *P* = 0.00054), PLAUR (HR = 1.4, *P* = 0.00920), SERPINE1 (HR = 1.5, *P* = 0.00250) and SPP1 (HR = 1.3, *P* = 0.04600) (Fig. 10). In addition, GSE102349 with 113 NPC patients was downloaded to re-evaluate the effect of the five core genes on PFS of NPC patients and the clinical parameters of 113 patients were presented in Table 4. We found that only high expression of SPP1 was significantly related with poor PFS of NPC patients (*P* = 0.04476, Fig. 11A). Then, the diagnostic value of the 10 core genes in NPC was evaluated via GSE12452 dataset and we found that the area under the curve (AUC) of nine core genes (FN1, MMP1, MMP3, PLAU, PLAUR, SERPINE1, SPP1, COL8A1, COL10A1) were all more than 0.8 (*P* < 0.05, Fig. 11B, Table 5) and among them, the AUC of FN1 and PLAU were close to 1. These results indicated that the nine genes might be diagnostic markers of NPC.

## Discussion

NPC is a malignant tumor originating from epithelium of nasopharynx and the most powerful predictor for prognosis is the stage of NPC. The 5-year survival rate of patients could decrease with the increase of the stage of NPC 11. However, NPC is generally diagnosed at a locoregionally advanced stage 12. Therefore, early diagnosis is beneficial to prolong the survival time of NPC patients. It is key to explore the pathogenesis of NPC and search for molecular markers related to early diagnosis of NPC.

In our study, we identified 314 DEGs of NPC totally based on GSE40290 and GSE53819. These genes were found to be mainly related to signal transduction, extracellular matrix organization and cell adhesion based on GO analysis. For KEGG analysis, we found that NF-κB signaling pathway, pathways in cancer and B cell receptor signaling pathway were mainly enriched pathways, which were mostly associated with the development of cancer. NF-κB is a nuclear transcription factor and participates in regulating the expression of multiple genes that are important for tumorigenesis, inflammation and autoimmune diseases 13. NF-κB signaling pathway mediated chronic inflammation might contribute to persistent EBV infection that further contributes to nasopharyngeal carcinogenesis 14-16. Then, 10 up-regulated core genes were obtained by constructing PPI network and gene expression level analysis in NPC, including FN1, MMP1, MMP3, PLAU, PLAUR, SERPINE1, SPP1, COL8A1, COL10A1 and COL17A1. Subsequently, the expression of SPP1 was found to be negatively correlated with overall survival and PFS of NPC patients by GEPIA and GSE102349 dataset, which indicated that SPP1 might be a prognostic marker for NPC. In addition, based on ROC curve analysis of GSE12452, nine genes (FN1, MMP1, MMP3, PLAU, PLAUR, SERPINE1, SPP1, COL8A1, COL10A1) might serve as diagnostic markers of NPC.

Fibronectin 1 (FN1) is a member of the ligand glycoprotein family, which is expressed in many kinds of cells and participates in cellular adhesion and migration process 17. The high expression of FN1 suppressed apoptosis of NPC cells by NF-κB pathway, which might lead to cell migration 18. MMP1 and MMP3 are members of matrix metalloproteinases (MMPs) 19. MMP1 belongs to collagenase and Song et al. found that MMP1 was over-expressed in NPC and it could promote cell proliferation, suppress cell apoptosis and increase the resistance to 5-fluorouracil in NPC 20. MMP3 was induced by Zta, which was a lytic transactivator of Epstein-Barr virus, and promoted cell migration and invasion in NPC 21. PLAU is urokinase-type plasminogen activator and PLAUR is the receptor of PLAU. They were also reported to promote NPC cell proliferation and invasion through different signaling pathways, such as Notch signaling pathway and JAK-STAT pathway 22, 23. Serpin Family E Member 1 (SERPINE1) is a fibrinolytic inhibitor, which plays a crucial role in various tumors 24. Sang et al. found that the transcription factor TEL2 suppressed metastasis of NPC by down-regulating SERPINE1 and the up-regulation of SERPINE1 could promote metastasis of NPC 25. Secreted phosphoprotein 1 (SPP1) is a phosphorylated extracellular matrix protein, which is a metastasis-associated gene 26. In NPC, the high expression of SPP1 resulted in shorter survival time of NPC patients and SPP1 could improve the ability of cell proliferation and migration 27, 28. COL8A1, COL10A1 and COL17A1 are all members of collagen family. Collagen is the main structural protein in the extracellular space, which participates in the formation of tumor microenvironment and promotes metastasis of tumor 29. The expression of COL8A1, COL10A1 and COL17A1 were found to be significantly elevated in many different tumor types 30-34 and they were all tumor-related genes. COL8A1 could improve the ability of cell proliferation and invasion, and the over-expression of COL8A1 was associated with the lymphatic metastasis of hepatocellular carcinoma 35. COL10A1 was proved to promote metastasis by inducing epithelial-mesenchymal transition in gastric cancer 31 and the over-expression of COL10A1 had a negative effect on the prognosis of patients in colorectal cancer 32. COL17A1 might be related to cellular motility and adhesion, which had the potential to promote the progress of tumor 36. However, the role of COL8A1, COL10A1 and COL17A1 in NPC has not been studied. In our study, the three novel genes were validated to be significantly up-regulated in NPC tissues and cells. Furthermore, the ROC curve analysis showed that COL8A1 and COL10A1 had good diagnostic value for NPC and they might be diagnostic markers for NPC. DEGs were mainly enriched in extracellular matrix related biological processes in our study and the three novel genes are collagen, which is the main component of extracellular matrix and participates in the formation of tumor microenvironment 29. These results suggested that they might play a regulatory role in NPC by altering tumor microenvironment and regulating cellular adhesion, which needs further experimental explorations to validate in the future.

Furthermore, the TF-microRNA coregulatory network of the 10 core genes was constructed and there were 33 TFs and 17 microRNAs in the network. We found that Jun could regulate the most core genes, including MMP1, MMP3, PLAU, PLAUR, SERPINE1 and SPP1. Cellular Jun (c-Jun) is an essential member of the activating protein-1 (AP-1) transcription factor family, which can induce oncogenic transformation 37. AP-1 is a dimer composed of c-Jun and c-Fos 38. The AP-1 binding sites in the promoter of MMP1 and MMP3 were essential for the expression of the two genes 39, 40, and the combination of them promoted the expression of MMP1 in osteosarcoma 39. In addition to be induced by AP-1, PLAU and PLAUR could also up-regulate the expression of AP-1, which promoted the invasion of trophoblast 41. c-Jun could also induce the expression of SERPINE1 in human hepatoma cell line 42 and might cause the migration of endothelial cell 43. SPP1 was found to be regulated by c-Jun N-terminal kinase (JNK) signaling pathway and could promote the lung metastasis of breast cancer 44. There were four microRNAs (hsa-miR-127-5p, hsa-miR-144, hsa-miR-204, hsa-miR-802) could regulate three core genes in the network (Fig. 9B). hsa-miR-127-5p could combine with the 3'UTR of SPP1 to inhibit the proliferation of chondrocyte in osteoarthritis 45. Zhang et al. discovered that hsa-miR-144 could combine with 3'UTR of FN1 to inhibit cell invasion in papillary thyroid cancer 46. hsa-miR-204 was down-regulated in NPC cells as well as tissues and it could inhibit cell invasion and radioresistance by regulating target gene in NPC 47. The regulatory relationship between MMP3, PLAUR, SERPINE1 and hsa-miR-204 in the network have not been reported, but the three core genes were predicted as target genes of hsa-miR-204 by miRTarBase database. hsa-miR-802 is also a tumor-related microRNA and it could inhibit the development of many tumors like gastric cancer and ovarian cancer 48, 49. However, whether hsa-miR-802 can regulate the three core genes (FN1, PLAUR, SPP1) needs further study to validate. We evaluated the expression of the five key elements in NPC cells and we found that c-Jun was significantly up-regulated in NPC cells, and three microRNAs (hsa-miR-127-5p, hsa-miR-204, hsa-miR-802) were significantly down-regulated in NPC cells (*P* < 0.05). These results indicated that these key elements might be involved in the upstream mechanism that regulates core genes. Core genes might promote the development of NPC through transcriptional regulation of c-Jun and the three microRNAs might inhibit the progress of NPC by silencing the expression of their corresponding core genes.

There were several studies have previously identified many DEGs of NPC through bioinformatical analysis 50-52. Compared with previous studies, the advantages of our study may be summarized as follows: Firstly, these previous reports have only screened core genes for NPC and they did not further evaluate the clinical value of core genes for NPC. Due to the lack of clinical data of NPC in public databases, very few studies have evaluated the clinical value of core genes for NPC. Han et al. only explored the effect of their core genes on prognosis of NPC patients based on the clinical data of head and neck squamous cell carcinoma (HNSCC) in public database 53. However, the clinical features of NPC and HNSCC are different. Therefore, the validation based on HNSCC was not specific for NPC. In our present study, gene expression datasets of NPC from GEO database, including GSE102349 and GSE12452, were used for prognostic and diagnostic analysis of core genes in NPC, respectively. Therefore, the evaluation of core genes related clinical value for NPC in our present study were more reliable than these previous studies and we also obtained several prognostic and diagnostic biomarkers for NPC. Secondly, multiple previous reports have only selected key biomarkers of NPC at mRNA level and they did not further explore upstream molecules that might regulate core genes 53-55. In our present study, we constructed TF-microRNA regulatory network of core genes. The expression of the key elements in network, including c-Jun, hsa-miR-127-5p, hsa-miR-204, hsa-miR-802, were also validated in NPC cells and they might be involved in the upstream mechanism that regulate core genes, which provides a new direction to study the pathogenesis of NPC. Thirdly, three novel genes that have not been studied in NPC were identified in our study, including COL8A1, COL10A1 and COL17A1. The three core genes might be novel molecules that participated in nasopharyngeal carcinogenesis, which are worthy for further study.

In conclusion, the purpose of the present study was to identify DEGs and potential pathways for NPC through bioinformatical methods. 314 DEGs were identified totally and several biological processes and signaling pathways that these DEGs mainly enriched in, including extracellular matrix related biological processes, NF-κB signaling pathway, pathways in cancer, B cell receptor signaling pathway and ECM-receptor interaction, might be critical for nasopharyngeal carcinogenesis. 10 up-regulated core genes were screened out and the upstream TFs and microRNAs of the 10 core genes, including c-Jun, hsa-miR-127-5p, hsa-miR-204 and hsa-miR-802, might participate in nasopharyngeal carcinogenesis by regulating corresponding core genes. Prognostic analysis revealed that the expression of SPP1 was negatively correlated with overall survival and PFS of NPC patients. Diagnostic analysis revealed that these core genes, including FN1, MMP1, MMP3, PLAU, PLAUR, SERPINE1, SPP1, COL8A1, COL10A1, had high diagnostic value for NPC. Although several prognostic and diagnostic markers of NPC were screened out in our study, further experimental explorations to research the function of the candidate genes are still needed in the future.

## Figures and Tables

**Table 1:** The sequences of all primers for qRT-PCR

Genes	Forward primer (5'-3')	Reverse primer (5'-3')
FN1	TGATCACATGGACGCCTGC	GAGTCAAGCCGGACACAACG
MMP1	AAGAATGATGGGAGGCAAGT	GGTTTCAGCATCTGGTTTCC
MMP3	AGTCTTCCAATCCTACTGTTGCT	TCCCCGTCACCTCCAATCC
PLAU	AACTCTGCCACTGTCCTTC	CGGTTGTCTGGGTTCCTG
PLAUR	GCCTTACCGAGGTTGTGTGT	CATCCAGGCACTGTTCTTCA
SERPINE1	AGTGGACTTTTCAGAGGTGGA	GCCGTTGAAGTAGAGGGCATT
SPP1	TGCTGGTTGTAGACCCCAAAAG	CAGGGAGTTTCCATGAAGCCAC
COL8A1	CCACTTGCAGTATGGCAAAGA	CCCTCGTAAACTGGCTAATGGTA
COL10A1	AAGAATGGCACCCCTGTAATGT	ACTCCCTGAAGCCTGATCCA
COL17A1	CTGACTTTGCTGGAGATCTGG	TAGGCCATCCCTTGCAGTAG
c-JUN	AAAGGAAGCTGGAGAGAATCG	TGTTTAAGCTGTGCCACCTG
hsa-miR-127-5p	GCCGAGCTGAAGCTCAGAGG	CTCAACTGGTGTCGTGGA
hsa-miR-144	ACACTCCAGCTGGGTACAGTA	CTCAACTGGTGTCGTG
hsa-miR-204	CTGTCACTCGAGCTGCTGGAATG	ACCGTGTCGTGGAGTCGGCAATT
hsa-miR-802	CAGTAACAAAGATTCATCC	GAACATGTCTGCGTATCTC
GAPDH	ACAACTTTGGTATCGTGGAAGG	GCCATCACGCCACAGTTTC
U6	CTCGCTTCGGCAGCACATATACT	ACGCTTCACGAATTTGCGTGTC

**Table 2:** The GO and KEGG pathway analysis of 314 DEGs in NPC

Category	Term	Count	%	*P* Value	FDR
GOTERM_BP_DIRECT	GO:0007155~cell adhesion	29	0.064215	1.20E-08	2.04E-05
GOTERM_BP_DIRECT	GO:0030574~collagen catabolic process	10	0.022143	1.71E-06	0.002913
GOTERM_BP_DIRECT	GO:0030198~extracellular matrix organization	16	0.035429	2.11E-06	0.003594
GOTERM_BP_DIRECT	GO:0050900~leukocyte migration	12	0.026572	1.05E-05	0.017855
GOTERM_BP_DIRECT	GO:0007165~signal transduction	42	0.093001	1.59E-05	0.026996
GOTERM_CC_DIRECT	GO:0005615~extracellular space	59	0.130644	1.50E-11	1.99E-08
GOTERM_CC_DIRECT	GO:0005576~extracellular region	57	0.126215	8.37E-08	1.11E-04
GOTERM_CC_DIRECT	GO:0031012~extracellular matrix	20	0.044286	5.79E-07	7.66E-04
GOTERM_CC_DIRECT	GO:0005887~integral component of plasma membrane	50	0.110715	7.09E-07	9.38E-04
GOTERM_CC_DIRECT	GO:0005886~plasma membrane	106	0.234716	1.00E-06	0.001323
GOTERM_MF_DIRECT	GO:0050839~cell adhesion molecule binding	8	0.017714	7.90E-05	0.113113
GOTERM_MF_DIRECT	GO:0005102~receptor binding	18	0.039857	1.06E-04	0.151684
GOTERM_MF_DIRECT	GO:0005178~integrin binding	9	0.019929	3.96E-04	0.565681
GOTERM_MF_DIRECT	GO:0005515~protein binding	171	0.378645	0.004543	6.314384
GOTERM_MF_DIRECT	GO:0005201~extracellular matrix structural constituent	6	0.013286	0.005372	7.425958
KEGG_PATHWAY	hsa04064: NF-kappa B signaling pathway	13	0.028786	4.62E-07	5.77E-04
KEGG_PATHWAY	hsa04662: B cell receptor signaling pathway	10	0.022143	2.12E-05	0.026444
KEGG_PATHWAY	hsa05200: Pathways in cancer	23	0.050929	6.18E-05	0.077161
KEGG_PATHWAY	hsa04512: ECM-receptor interaction	10	0.022143	1.36E-04	0.169313
KEGG_PATHWAY	hsa04640: Hematopoietic cell lineage	10	0.022143	1.36E-04	0.169313

**Table 3:** The KEGG pathway analysis of 25 core genes in NPC

Category	Term	Count	%	*P* Value	FDR
KEGG_PATHWAY	hsa04610: Complement and coagulation cascades	5	0.133120	6.37E-05	0.070385
KEGG_PATHWAY	hsa05200: Pathways in cancer	7	0.186368	0.001209	1.329008
KEGG_PATHWAY	hsa04062: Chemokine signaling pathway	5	0.133120	0.001949	2.134442
KEGG_PATHWAY	hsa05222: Small cell lung cancer	4	0.106496	0.002332	2.548887
KEGG_PATHWAY	hsa04064: NF-kappa B signaling pathway	4	0.106496	0.002581	2.817598

**Table 4:** Clinical characteristics of 113 NPC patients in GSE102349

Characteristics	Number of patients
**Age (years)**	
Median	45.5
25^th^ percentile	39
75^th^ percentile	54
**Gender**	
Male	86
Female	26
Unknown	1
**Smoking history**	
Smokers	32
Non-smokers	46
Unknown	35
**Histologic type**	
Non-keratinizing carcinoma, differentiated	85
Non-keratinizing carcinoma, undifferentiated	22
Unknown	6
**Clinical stage**	
Stage I	5
Stage II	2
Stage III	41
Stage IV	25
Unknown	40

**Table 5:** The diagnostic analysis of core genes in NPC based on GSE12452

Core genes	*P* value	AUC with 95% CI
FN1	< 0.0001	0.9839 (0.9490-1.0000)
MMP1	0.0003	0.8871 (0.7847-0.9895)
MMP3	< 0.0001	0.9516 (0.8889-1.0000)
PLAU	< 0.0001	0.9968 (0.9863-1.0000)
PLAUR	0.0043	0.8032 (0.6194-0.9870)
SERPINE1	0.0002	0.8968 (0.8017-0.9918)
SPP1	0.0009	0.8516 (0.6940-1.0000)
COL8A1	< 0.0001	0.9484 (0.8845-1.0000)
COL10A1	0.0003	0.8839 (0.7802-0.9875)
COL17A1	0.1917	0.6387 (0.4765-0.8009)

AUC: The area under the curve; CI: Confidence interval.

## Abbreviations

NPC: nasopharyngeal carcinoma
GEO: Gene Expression Omnibus
DEGs: differentially expressed genes
DAVID: Database for Annotation, Visualization and Integrated Discovery
GO: Gene Ontology
KEGG: Kyoto Encyclopedia of Genes and Genomes
BP: biological process
CC: cellular component
MF: molecular function
PPI: protein-protein interaction
STRING: Search Tool for the Retrieval of Interacting Genes
MCODE: Molecular Complex Detection
GEPIA: Gene Expression Profiling Interactive Analysis
TF: transcription factor
TCGA: The Cancer Genome Atlas
PFS: progression-free survival
ROC: receiver operating characteristic
AUC: area under the curve
